# Application of in-silico docking for green electrochemical quantification of prucalopride succinate in pharmaceutical, urine, and milk matrices

**DOI:** 10.1038/s41598-024-85001-y

**Published:** 2025-02-18

**Authors:** Ahmed R. Mohamed, Rania A. Sayed, Abdalla Shalaby, Hany Ibrahim

**Affiliations:** 1 Pharmaceutical Chemistry Department, Faculty of Pharmacy, Egyptian Russian University, Badr City, 11829 Cairo Egypt; 2 Analytical Chemistry Department, Faculty of Pharmacy, Zagazig University, Zagazig, 44519 Egypt

**Keywords:** Prucalopride succinate, Potentiometry, Cyclodextrin, Molecular modeling, Greenness assessment., Chemistry, Analytical chemistry

## Abstract

The development of ion-selective sensors as green and energy-efficient substitutes is a promising trend in today’s analytical techniques. So, three distinct polyvinyl chloride sensors modulated by ionophores of cyclodextrins (α-, β-, and γ-cyclodextrins) were built, and their composition was optimized for rapid in-situ estimation of prucalopride succinate in different matrices. The experimental work was backed up by applying molecular docking, whose results were consistent with the practical findings, offering a creative strategy to reduce the experiment’s costs and duration. According to findings, the α-cyclodextrin sensor outperformed the other proposed sensors in terms of Nernstian slope (56.87 mV/decade), linearity range (1 × 10^−6^ M – 1 × 10^−2^ M), and detection limit (7.50 × 10^−7^ M). For the first time and with minimal sample pre-treatment, prucalopride was quantified with excellent recoveries in dissolution media, human urine, and formula milk samples employing the proposed α-cyclodextrin sensor. The proposed three sensors were validated following IUPAC guidelines. The proposed technique, represented by the proposed three sensors, has the following merits: high sample throughput, quick analysis, high selectivity, wide linearity ranges, and low detection limits, making it apt for routine prucalopride assessment, either in QC centers or in bioavailability units, at the most affordable price. Concerning the statistical assessment, there weren’t palpable disparities in functioning between the suggested technique and the published one. Concerning green assessment, the proposed technique surpassed the published techniques in both GAPI and analytical eco-scale metrics.

**Supplementary Information:**

The online version contains supplementary material available at 10.1038/s41598-024-85001-y.

## Introduction

Constipation is a self-reported common gastrointestinal disorder that affects 30% of the world’s population, especially women and the elderly^1^. Constipation is typically marked by infrequent bowel movements, bloating, lumpy or hard stools, and straining, with some complications like anal fissures and hemorrhoids^2^. The aforesaid symptoms and complications significantly lower constipated patients’ life quality and burden them financially^3^. The mainstays of constipation treatment are dietary and lifestyle modifications, medication, and, rarely, surgery^4^. One of the most efficient medications for constipation, especially chronic idiopathic constipation, is prucalopride succinate (PCPS)^1^.

**Fig. 1:**
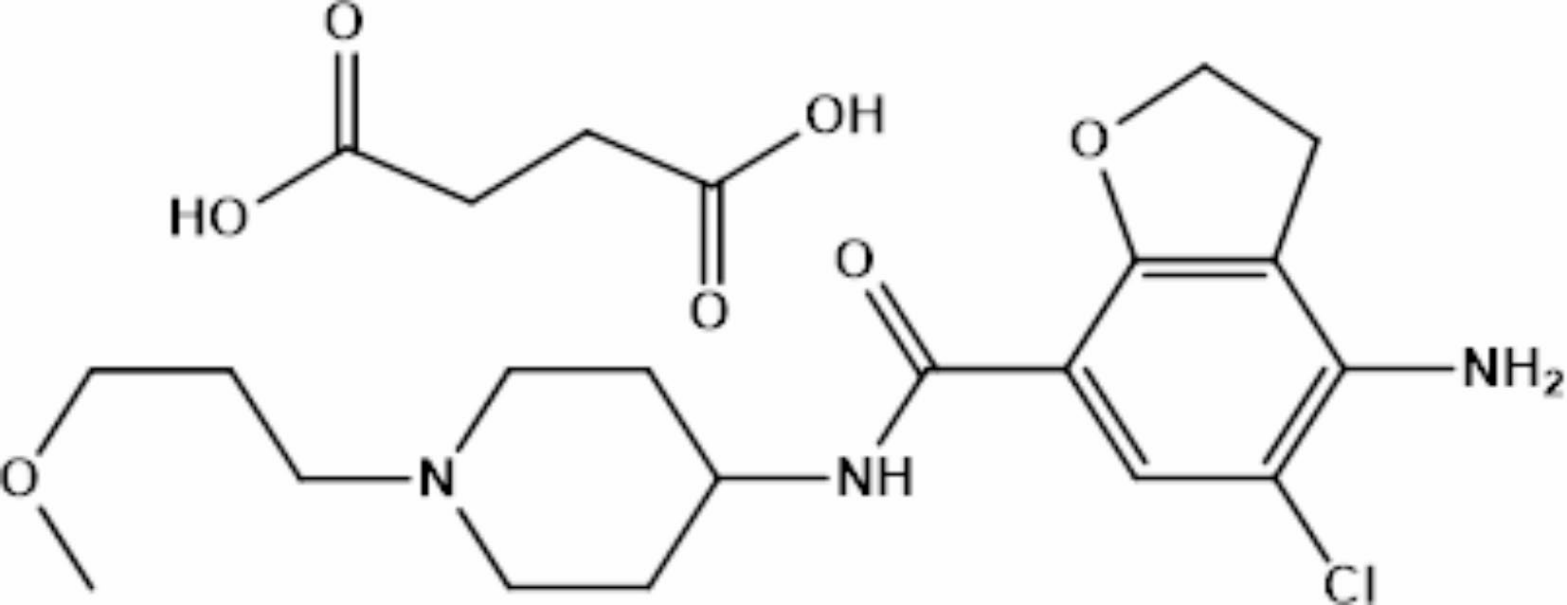
Chemical structure of prucalopride succinate.

PCPS (Fig. 1), a highly selective agonist for the 5-HT_4_ receptor, boosts intestinal motility (peristalsis) as an enterokinetic agent by triggering neural signaling^4^. It improves stool consistency, stool frequency, and straining^5^. In comparison to other 5-HT_4_ receptor agonists (e.g., cisapride and mosapride), PCPS is well-tolerated and safe, with no significant cardiovascular impacts or drug interactions^4,6^.

Previous reports discussed the determination of PCPS in different matrices by different techniques, including spectrophotometric^7–9^, spectrofluorimetric^10–12^, RP-HPLC-UV^13–16^, LC-MS/MS^17–19^, and electrochemical^20,21^ techniques. Even though some of these techniques offered good specificity and sensitivity, they were unable to apply quick in-situ detection of PCPS due to the bulky, expensive instruments and time-consuming procedures they used. These drawbacks mirrored our concerns about employing potentiometric techniques. Such techniques (potentiometric ones) have the merits of being straightforward, reasonably priced, delicate, eco-friendly, and reliable, allowing their usage in many fields. As well, potentiometric sensors relying on ion pairs are portable by nature, more selective, and have a faster response, making their assembly and design more accessible^22^. Further research revealed that ion-selective sensors (ISSs) relying on ionophores have significantly higher selectivity, responsiveness, stability, and lower detection limits (higher sensitivity) than those relying solely on ion pairs^22,23^.

Numerous studies have demonstrated the application of potentiometric ISSs to assess several drugs employing various modifiers (ionophores) of cyclodextrins according to the host-guest inclusion method^23,24^. The formation of inclusion complexes involves the emergence of non-covalent interactions among the host (cyclodextrins) and the guest (analyte)^25^. Molecular complexation, involving recognition and inclusion processes, is crucial for supramolecular chemistry and for the host-guest method. One approach of molecular complexation agents is cyclodextrin (CD) molecules, including α-, β-, and γ-CDs^26^. Cyclodextrins have distinctive toroidal structures with lipophilic (non-polar) cavities inside and hydrophilic (polar) surfaces with hydroxyl groups outside, allowing the formation of water-soluble inclusion complexes between CDs and many hydrophobic, poorly soluble analytes^24,26^.

This effort aims to design novel CD-modulated ISSs (PVC membrane sensors) for rapid in-situ estimation of PCPS in different matrices (commercial product, dissolution media, human urine, and formula milk samples). Additionally, molecular modeling (docking) was employed to study the properties of CDs-PCPS complexes and to substantiate the strong correlation between the practical and theoretical (in-silico docking) work for selecting the best sensor. The current study is unique in that it utilized the merits of both ion-exchanger and ionophores (CDs), as demonstrated by the anticipated electrodes’ performance.

This research presents the first in-situ quantification of PCPS with minimal sample pre-treatment and excellent recoveries in dissolution media, human urine, and formula milk samples. Moreover, the proposed technique offers the following merits: high sample throughput, quick green analysis, high selectivity, wide linearity ranges, and low detection limits, making it apt for routine PCPS assessment, either in QC centers or in bioavailability units, at the most affordable price.

Finally, two appraisal metrics, known as GAPI and eco-scale (ES), were introduced to appraise the proposed potentiometric technique’s greenness for the operated procedures, consumed chemicals, and solvents, exposing an outstanding greenness profile for this technique compared to the published techniques^15,20,21^.

## Experimental

### Solvents and materials

All materials or reagents utilized in this research were of analytical grade.

- The PCPS standard was an endowment from Mash Premiere Co^®^ (Cairo, Egypt). It obtained a 99.50% purity certification.
- Polyvinyl chloride (PVC), sodium tetraphenylborate (ST), dioctyl phthalate (DP), tetrahydrofuran, tributyl phosphate (TP), benzyl acetate (BA), α-, β-, and γ-CDs (Sigma-Aldrich, Germany).
- Magnesium stearate, BaCl_2_, NiCl_2_.6H_2_O, KCl, NH_4_Cl, NaCl, sucrose, glycine, urea, lactose, and glucose (Prolabo, USA).
- Starch, microcrystalline cellulose, phosphoric acid, glacial acetic acid, sodium hydroxide, and boric acid (Adwic, Egypt). Hence, Britton-Robinson buffer solutions (ranging from pH 2 to 12) were primed and then amended to the desired pH employing 0.20 M sodium hydroxide^27^.
- Phosphate buffer (5 × 10^−2^ M aqueous solution, pH = 6.80) (Adwic, Egypt).
- The water used throughout the experiment was bi-distilled.
- Healthy volunteers provided urine samples, which were then preserved at -20 °C until analysis.
- Infant formula milk, Similac 1^®^ was bought from the local store.

### Pharmaceutical formulation

Prucasoft^®^ tablets, made by Marcyrl Company (Egypt), Lot no. 2,232,381, are alleged to comprise 2.64 mg PCPS in a single tablet.

### Apparatus and software

- A digital pH 3510 ion-analyzer (Jenway, England) with Ag/AgCl double-junction reference electrode was utilized in conjunction with PVC-PCPS ISSs to conduct all potentiometric measurements at ambient temperature.
- pH glass electrode (Jenway, England) was employed for pH adjustment.
- Molecular operating environment (MOE^®^) software v2019.0102 was employed to perform the in-silico molecular docking studies between PCPS and CDs.
- USP dissolution device (Edition VK 7000, Paddle) was utilized for in-vitro dissolution tests.
- Sonicator (Edition WUC-A06H) was also utilized in this research.

### Standard solutions

Stock standard solution of PCPS (10^−2^ M) was primed by sonicating 486 mg of PCPS powder for 3 min in 80 mL buffer (Britton-Robinson) of pH 6 into a 100-mL calibrated flask. The volume was then filled up to the mark (100-mL) by the buffer. Serial dilutions were implemented to prepare different working solutions of PCPS varying from 1 × 10^−7^ M – 1 × 10^−3^ M employing the same buffer.

## Procedures

### Ion-pair (PCPS-ST) preparation

Equivalent amounts of 0.01 M solutions of both PCPS and ST were mingled, and the mixture was left overnight to allow complete precipitate coagulation. The resulting residue was filtrated and rinsed several times with bi-distilled water to remove the remaining free PCPS. Finally, the coagulated part (precipitate) was left to dry completely at ambient temperature (for 24 h).

### Preparation of PCPS-CDs-modulated PVC membranes

The desired PVC membranes were primed by adding PVC powder (190 mg), ion-pair (PCPS-ST precipitate) (8 mg), tetrahydrofuran (5 mL), and DP plasticizer (295 mg) to three 5-cm glass Petri dishes. A glass rod was employed to thoroughly mix the aforesaid ingredients before adding 10 mg of each ionophore (α-CD, β-CD, and γ-CD) separately. Afterward, all ingredients were mingled for five minutes employing a magnetic stirrer before being covered with filter papers. Finally, all mixtures were left all night to allow complete evaporation of tetrahydrofuran and drying of the PVC membranes at ambient temperature.

### Electrode assembly

**Fig. 2:**
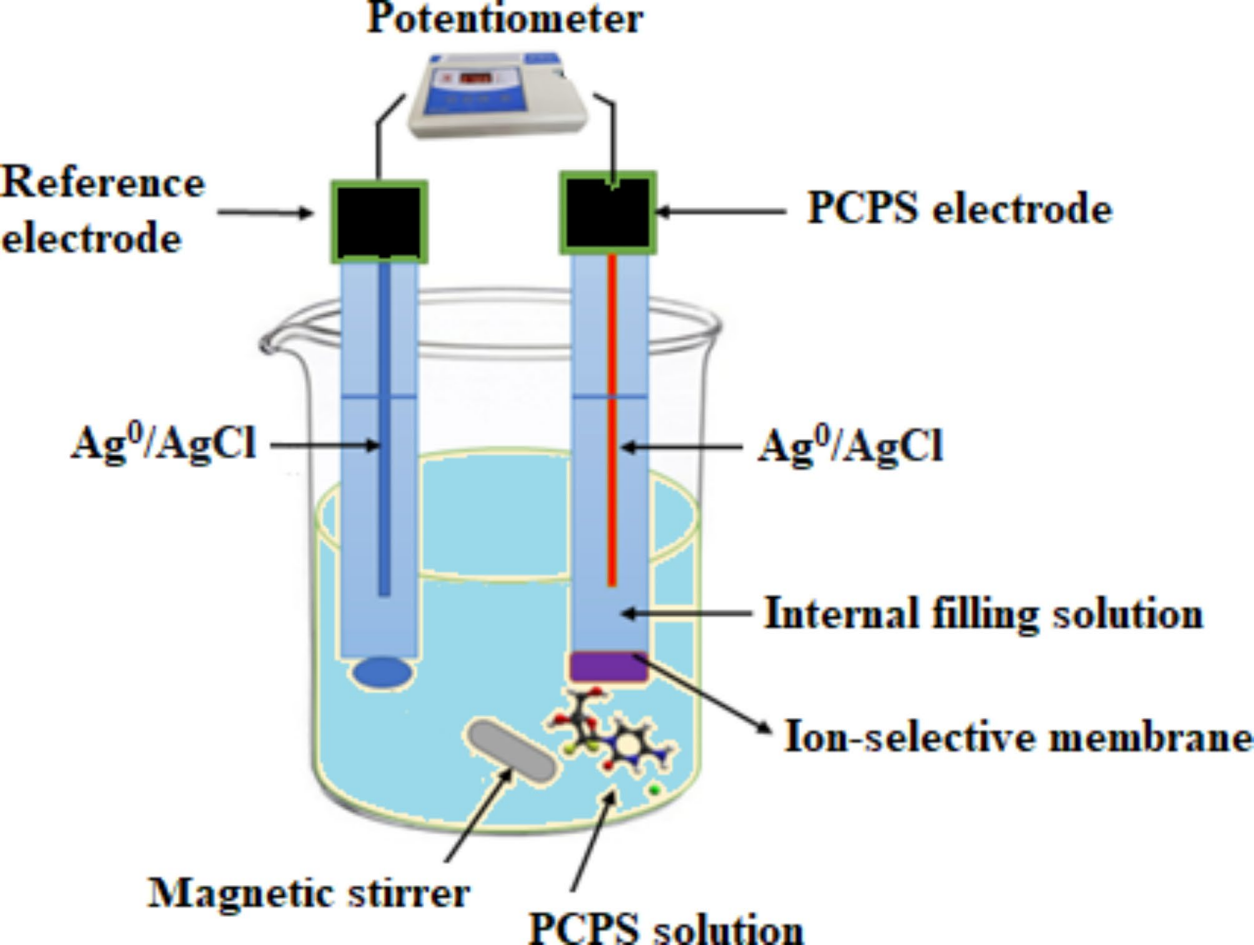
Potentiometric measurement system employing a double junction reference sensor and PCPS indicator sensor.

The proposed sensors were created by slicing three disks of 1-cm diameter using a scalpel from the modulated PVC membranes. These disks were glued with tetrahydrofuran to a Tygon tube’s tip connected to the end of each glass electrode. Equivalent amounts of 0.01 M solutions of both PCPS and KCl (as the internal solution) were conveyed to each glass electrode. Afterward, an Ag/AgCl wire was dunked into each electrode’s internal solution (as the internal reference electrode) and externally connected to the potentiometer. Conditioning of the proposed sensors was carried out by soaking each one for 24 h in a 0.01 M PCPS standard solution. Figure 2 depicts the design of the proposed sensors.

### Calibration curves construction

The proposed sensors were calibrated by dipping each one in parallel with the external reference electrode (Ag/AgCl reference electrode) into standard solutions of PCPS varying from 1 × 10^−7^ M – 1 × 10^−2^ M. After stabilization, the potential readings (in mV) were recorded and then graphed versus the respective –log concentrations of PCPS. Finally, the regression equations were derived from the calibration plots to compute the unknown concentrations.

### Pharmaceutical application

Five tablets of Prucasoft^®^, each containing 2.64 mg of PCPS, were weighed precisely, crushed finely in a mortar, and homogeneously mixed. A precisely weighed section of the crushed tablets, equivalent to 2.64 mg PCPS (one tablet) was relayed into a 100-mL calibrating flask comprising 70 mL buffer (Britton-Robinson) of pH 6. After ten minutes of sonication, the solution volume was corrected by utilizing the same buffer till the mark (100-mL). The proposed sensors, connected to the external reference electrode, were employed to record the response (potential) in mV. Finally, the nominal content of PCPS in its formulation, besides its pure concentrations in M (after executing the standard addition protocol), was estimated employing each proposed sensor’s regression equation.

### Dissolution testing

As per the FDA’s guides^28^, the USP device-II (Paddle) was utilized to try the dissolution of PCPS from Prucasoft^®^ tablets at 37.00 ± 0.50 °C with a 75.00 RPM stirring speed. The dissolution medium involved 0.50 L phosphate buffer (pH 6.80, 50 mM). Afterward, the proposed sensor (PCPS-α-CD-PVC) together with the external reference electrode was dunked into the tested solution (dissolution medium). The potential readings (in mV) were recorded at time intervals from 5 min to 45 min. Finally, the proposed sensor’s regression equation was exploited to compute PCPS’s release percentages and to construct its dissolution profile.

### Application to human urine and formula milk samples

Into two sets of five calibrated flasks (25-mL), 5.00 mL aliquots of human urine or reconstituted formula milk samples were spiked with various standard aliquots of PCPS standard solution (10^−2^ M). Each solution volume was adjusted employing the buffer (Britton-Robinson) of pH 6 till the mark (25-mL) to get ultimate concentrations varying from 1 × 10^−6^ M – 1 × 10^−4^ M. All samples of urine or milk were shaken thoroughly after volume adjustment for one minute and then conveyed separately into glass beakers (50-mL). Afterward, the proposed sensor (PCPS-α-CD-PVC) together with the external reference electrode was dunked into the prepared samples. After stabilization, the potential readings (in mV) were recorded and then graphed versus the respective –log concentrations of spiked PCPS. The proposed sensor was rinsed with the same buffer between measurements. Finally, the regression equations (for urine and milk) were derived from the calibration plots to compute the unknown concentrations.

### Molecular docking

To support the aforesaid practical results, a molecular docking experiment was conducted on the inclusion complexes of PCPS and the ionophores (CDs) employing MOE^®^ software. The CDs were employed as selective receptors for PCPS (the ligand). The CDs were obtained as 3D crystal structures from the RCSB protein data bank, then isolated from their respective sources (Table S1), and equipped with dummy atoms for the docking experiment. While the 3D structure of PCPS was built employing the builder function in MOE^®^ software. Afterward, the isolated CDs were primed by adding hydrogen atoms (protonation) at pH 6, adjusting the partial charges, and minimizing their energies as per the MOE protocol of energy minimization. PCPS was primed similarly and then processed by the conformations search function to provide all potential conformations of PCPS, thus improving docking scores. The conformations search results of PCPS were stored as a database, ready for the docking experiment with each proposed ionophore. The docking experiment was implemented employing the MOE’s default docking protocol, and the docking scores were computed as per the London dG scoring function. Finally, the positions resulting from the docking experiment were classified based on their docking scores, and the best energy position was selected.

## Results and discussion

### Optimization of the sensors’ composition

Indeed, the sensitivity, selectivity, and linearity of ISSs are significantly affected by the membrane’s inherent components, particularly the plasticizers and ion pairs. Hence, different kinds and quantities of the membrane’s inherent components were tried to maximize the membrane’s functionality (potential responsiveness). The earlier study^26^ reported that plasticizers influence the membrane’s potential responsiveness owing to their dielectric constants. So, three sensors were fabricated employing three different plasticizers, including DP, BA, and TP. According to the outcomes (Table S2), the best-fabricated sensor was the one including DP as a plasticizer. Because the ion-pair complex is critical for membrane performance and sensor response, the impacts of various amounts of PCPS-ST association (ion-pair complex) in the membrane were investigated while keeping the other components unchanged (Table S2).

Despite having a long lifespan, the PVC membrane sensor performed poorly (had a subpar response), according to the outcomes in Table S2. Hence, three sensors were constructed, each containing 10 mg of α-, β-, or γ-CD as the ion-pair enhancer while keeping the other components unchanged. The findings of the three sensors, including linear ranges (M), correlation coefficients, Nernstian slopes, and LODs, were summarized in Table S2. For the upcoming experiments, the membrane sensor comprising 190 mg PVC, 8 mg PCPS-ST, 10 mg α-CD, and 295 mg DP was selected owing to its superior performance compared to the other sensors (Table S2).

#### pH effect

To provide the ideal trial condition, the influence of changing pH from 2 to 12 on the potential readings of the tested sensors was investigated by employing PCPS solutions of 10^−4^ M and 10^−3^ M. The pH profiles were established by plotting the potential readings versus each pH change (Fig. S1). The tested sensors showed stable potential readings in the pH range (4–8), Fig. S1. Below pH 4, the potential readings increased gradually due to protons’ interference (competition of hydronium ions of higher mobility with prucalopride ions in the membrane). In contrast, above pH 8, the potential readings decreased gradually due to the deprotonation of prucalopride ions and the formation of prucalopride base^20^.

#### Soaking time effect

The freshly prepared sensors were soaked into a 0.01 M PCPS standard solution to induce the formation of a thin gel layer at the modulated PVC membranes’ surfaces, where ion diffusion and exchange occur. Different soaking times from 0 to 36 h were tested. The potential readings (in mV) were recorded after each soaking time and then graphed versus the respective –log concentrations of PCPS. Finally, the slope values were computed from the calibration plots. The optimal soaking time that yielded the best responses for analyzing PCPS employing all proposed sensors was 24 h (Fig. S2).

#### Dynamic response time, stability, and reversibility

**Fig. 3:**
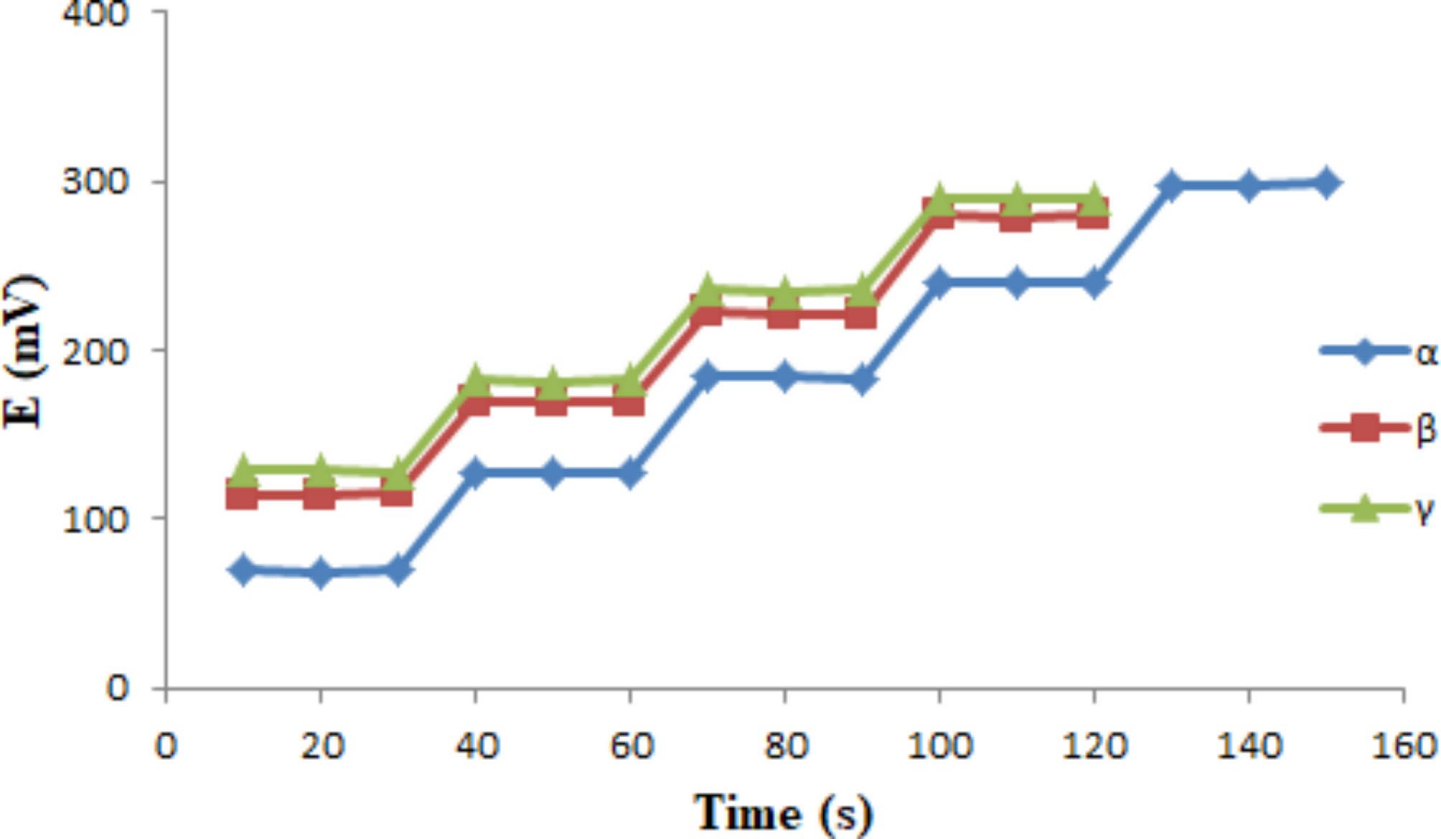
The response time curves of the suggested three sensors using PCPS solutions (1 × 10^−6^ M to 1 × 10^−2^ M) for the PCPS-α-CD-PVC sensor and (1 × 10^−5^ M to 1 × 10^−2^ M) for both sensors, PCPS-β-CD-PVC and PCPS-γ-CD-PVC.

For the three proposed sensors, the dynamic response times were assessed by monitoring the time needed to attain the steady-state (limiting) potential within ± 1 mV after a 10-fold increase in PCPS concentration. Figure 3 shows that the proposed sensors attained equilibrium potential within 10 s.

The stability (lifetime) of the proposed sensors was assessed by periodically creating calibration curves and investigating their parameters (slopes) for 5 successive weeks, as previously discussed in “Calibration curves construction”. Results showed that the proposed sensors had a 28-day lifespan, during which the potential variations didn’t exceed ± 2 mV.

Concerning the proposed sensors’ reversibility, the potentials of 1 × 10^−5^ M and 1 × 10^−3^ M PCPS solutions were alternately recorded every 5 min. Figure S3 shows good reversibility of the proposed sensors with rapid and steady responses that were within ± 1 mV of the initially recorded responses.

#### Molecular docking (in-silico study)

Molecular docking was employed to support the practical findings and to forecast how the guest (PCPS) and host (CDs) molecules would fit, align, and interact with one another. The docking trials divulged that PCPS interacted successfully with each suggested ionophore (α-, β-, and γ-CDs), forming inclusion complexes with docking scores consistent with the three proposed sensors’ Nernstian responses (slopes), Fig. S4. The docking scores were ^−^9.27, -8.66, and ^−^7.71 kcal/mol for α-, β-, and γ-CDs, in order. The smallest score (more negative score, -9.27 kcal/mol for α-CD) indicates the strongest binding interaction with PCPS, and vice versa. Hence, the best sensor for in-situ detection and/or quantification of PCPS was the PCPS-α-CD-PVC sensor.

### Method validation

The following IUPAC regulations^29^ were pursued to value the suggested potentiometric technique.

#### Linearity and range

**Fig. 4:**
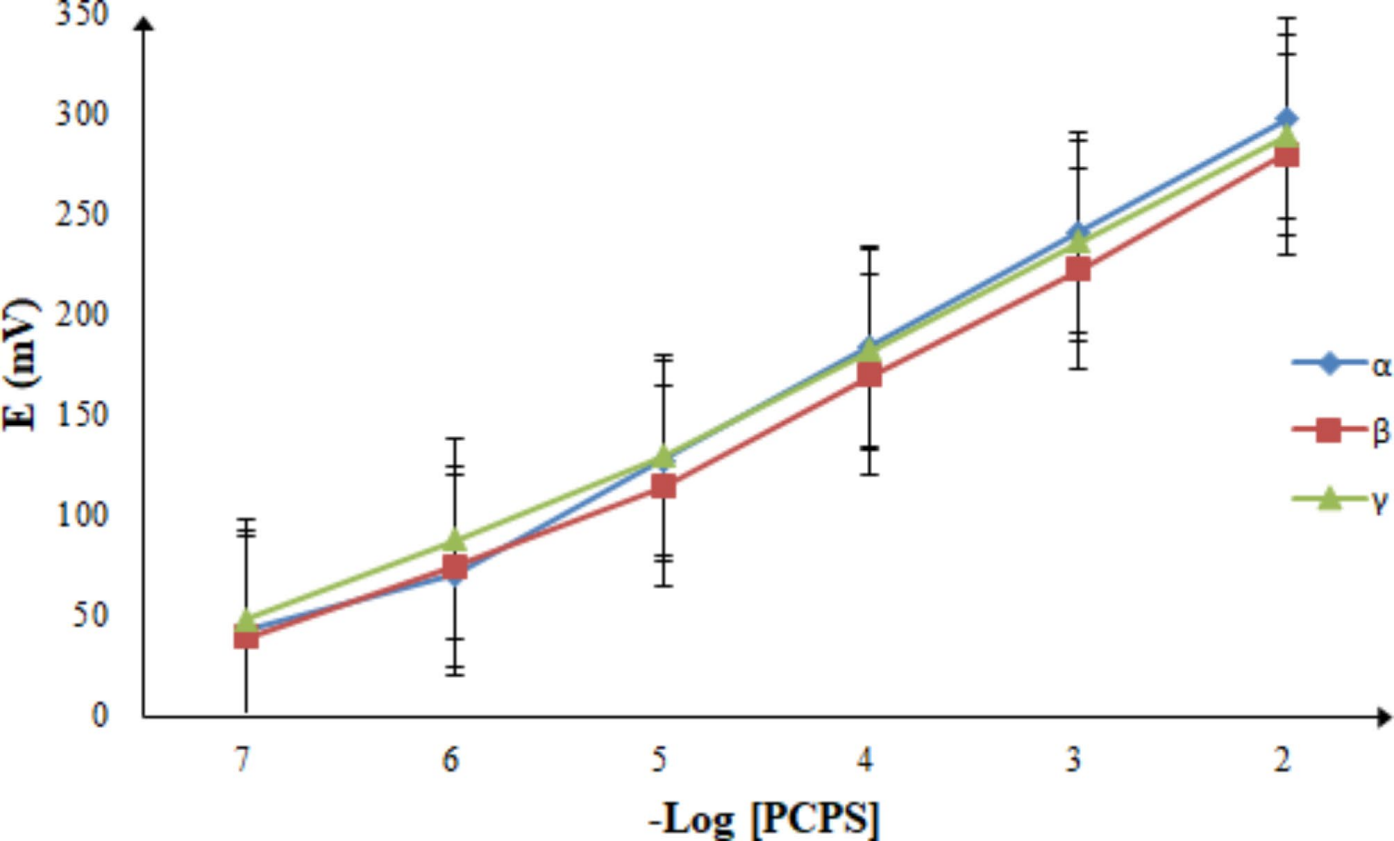
Potential calibration profiles for the suggested three sensors.

Pursuant to the ideal circumstances, the linear range was assessed by recording the potential readings of different concentrations of PCPS in triplicate. Good linearities were established for the three proposed sensors between the average potentials and the respective –log concentrations of PCPS (Fig. 4). The linearity data for each proposed sensor was recorded in Table 1.

**Table 1:** Analytical performance data of the suggested three sensors for the determination of PCPS.

Parameters	Sensors		
	PCPS-α-CD-PVC	PCPS-β-CD-PVC	PCPS-γ-CD-PVC
Linearity range (M)	1 × 10^−6^ – 1 × 10^−2^	1 × 10^−5^ – 1 × 10^−2^	1 × 10^−5^ – 1 × 10^−2^
Slope (mV/decade)	56.87	54.89	53.78
Intercept (mV)	410.82	388.69	396.64
Correlation coefficient (r )	0.9999	0.9998	0.9997
LOD (M)	7.50 × 10^−7^	7.10 × 10^−6^	7.20 × 10^−6^
Working pH range	4–8	4–8	4–8
Response time (s)	10	10	10
Stability (weeks)	4	4	4

#### Limit of detection

The detection limits (LOD) of PCPS were computed as per IUPAC guidelines at the intersection points of the calibration plots’ extrapolated lines. Table 1 shows the detection limit of each proposed sensor.

#### Accuracy and precision

The accuracy & the two categories of precision (repeatability and intermediate precision) were valued by scrutinizing (in triplicate) three crude concentrations casing all PCPS’s linearities by the three proposed sensors. On one day, the examination was executed for repeatability, and on three days in a row, for intermediate precision. Table S3 displays recovery% ± SD for accuracy findings and RSD% for precision findings. The aforementioned findings, besides the Er%, were acceptable, proving the proposed technique’s high levels of precision, reliability, and accuracy.

#### Selectivity

As per IUPAC guidelines, the separate solutions approach was employed to investigate the proposed sensors’ selectivity. The selectivity coefficients (K^Pot^) for the interfering substances were estimated by the separate solutions approach (Table S4). The K^Pot^ values were computed employing this equation^30^:

$$\:-log\:{K}_{Primary\:ion,\:\:interferent}^{Pot}=\:E1\:\--\:E2/S$$

where *E*_*1*_ is the response recorded in a 1 × 10^−3^ M PCPS solution (primary ion), *E*_*2*_ is the response recorded in a 1 × 10^−3^ M interferent solution, and *S* is the investigated sensor’s slope (mV/decade).

The outcomes in Table S4 reveal the proposed sensors’ high selectivity for PCPS without significant intrusion from the common interfering substances (owing to the low K values).

#### Robustness

The proposed technique’s robustness was valued (as per ICH guidelines^31^) by inspecting the sway of intended small tweaks in the ideal statuses on the proposed technique’s analytical functioning. The intended tweaks were in pH (6.00 ± 0.20) and soaking time (24 h ± 1 h). Finally, the small tweaks in the aforesaid factors disclosed trifling variances in recovery proportions relative to the ideal statuses, demonstrating the proposed technique’s robustness and reliability (Table S5).

### Method applications

#### Pharmaceutical application

Without extraction or derivatization, PCPS was quantified successfully in its product (Prucasoft^®^ tablets) by employing the proposed three sensors (Table 2). The listed findings in the aforementioned table were decent and closely resembled the formulation’s labeled PCPS quantity without interjecting with the formulation’s additives, demonstrating the proposed sensors’ appositeness for regular quantifying PCPS in QC labs. The proposed technique’s validity was inspected by executing the standard addition protocol, generating decent recovery proportions (Table 2).

**Table 2:** Determination of PCPS in Prucasoft^®^ tablets^a^ using the suggested three sensors and application of standard addition technique.

Suggested sensors	Taken (M)	Recovery% ± SD^b^	Standard addition		
			Added (M)	Found (M)	Recovery%
PCPS-α-CD-PVC	5.43 × 10^−5^	99.23 ± 1.16	1 × 10^−5^	1.01 × 10^−5^	101.10
			5 × 10^−5^	4.98 × 10^−5^	99.60
			1 × 10^−4^	0.99 × 10^−4^	99.20
			5 × 10^−4^	5.01 × 10^−4^	100.24
			5 × 10^−3^	4.97 × 10^−3^	99.42
			Mean ± SD		99.91 ± 0.77
PCPS-β-CD-PVC		99.68 ± 0.94	1 × 10^−5^	0.99 × 10^−5^	99.10
			5 × 10^−5^	5.02 × 10^−5^	100.46
			1 × 10^−4^	1.01 × 10^−4^	101.40
			5 × 10^−4^	4.96 × 10^−4^	99.28
			5 × 10^−3^	4.92 × 10^−3^	98.40
			Mean ± SD		99.73 ± 1.19
PCPS-γ-CD-PVC		98.70 ± 1.37	1 × 10^−5^	1.01 × 10^−5^	101.60
			5 × 10^−5^	5.04 × 10^−5^	100.80
			1 × 10^−4^	0.98 × 10^−4^	98.90
			5 × 10^−4^	4.95 × 10^−4^	99.02
			5 × 10^−3^	5.03 × 10^−3^	100.64
			Mean ± SD		100.19 ± 1.18

^a^Batch number (2232381).

^b^Mean of three determinations.

#### Dissolution testing (DT)

DT is an imperative action for the QC of pharmaceuticals and for prophesying the meticulous timing of active ingredients’ dose conveyance to patients^32,33^. Hence, DT establishes a linkage between in-vivo and in-vitro patterns. In this work, DT was executed on Prucasoft^®^ tablets to examine PCPS release from its formulation. To construct PCPS’s dissolution profile, the release proportions were reckoned by using the proposed sensor (PCPS-α-CD-PVC), then graphed against time intervals (minutes) (Fig. S5). It took 30 min to dissolve 99.11% of the Prucasoft^®^ tablets. Concerning this application (DT), the proposed sensor outperformed other analytical techniques (spectroscopic and chromatographic techniques) in terms of allowing continuous potential recording without requiring sample withdrawal at time intervals, sample filtration, or volume correction.

#### Human urine and formula milk samples

The earlier study in humans^34^ revealed significant excretion of PCPS into breast milk and urine (approximately 60% of the prescribed dose was excreted as prucalopride into the urine). The high delicacy (referring to small LOD values) accomplished by the suggested sensor (PCPS-α-CD-PVC) was exploited for PCPS estimation in human urine and formula milk (the closest substitute for human milk) samples with minimal pre-treatment (Table 3). The recoveries recorded in Table 3 divulge that the suggested sensor can be applied to studies on PCPS bioequivalence without any interjection from biological fluids’ matrices.

**Table 3:** Validation parameters for the quantification of PCPS by the suggested sensor (PCPS-α-CD-PVC) in human urine and formula milk samples.

Parameters	Urine	Milk
Concentration range (M)	1 × 10^−6^ – 1 × 10^−4^	
Correlation coefficient (r)	0.9996	0.9997
Slope (mV/decade)	56.76	56.64
Intercept (mV)	413.69	414.87
LOD (M)	7.90 × 10^−7^	8.30 × 10^−7^
Accuracy* (mean ± SD)	100.55 ± 1.56	99.86 ± 1.37
Intra-day precision (RSD%)	1.26	1.44
Inter-day precision (RSD%)	1.29	1.52

*Mean of five determinations.

## Greenness assessment

Two appraisal metrics, known as GAPI and ES, were introduced to appraise the published^15,20,21^ and proposed techniques’ greenness for the executed procedures, consumed chemicals, and solvents.

**Fig. 5:**
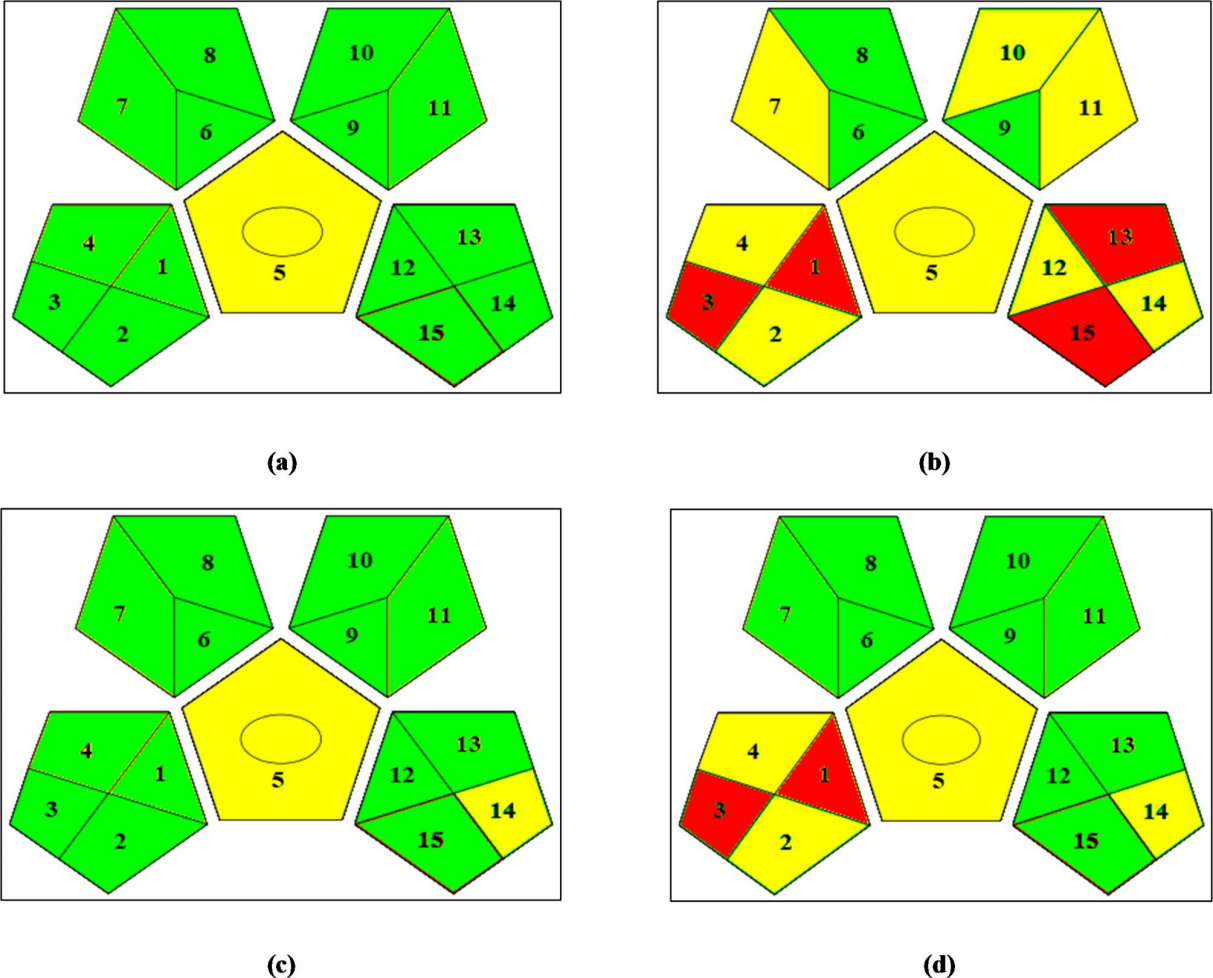
Greenness assessment profiles using the GAPI metric for (**a**) the suggested potentiometric approach, (**b**) the reported^15^ approach, (**c**) the reported^20^ approach, and (**d**) the reported^21^ approach.

Concerning the GAPI metric, the greenness profiles for the published and proposed techniques were created, disclosing the proposed technique’s supremacy from the greenness standpoint (Fig. 5). Concerning the ES metric, the ES scores for the published and proposed techniques were calculated (Table S6), exposing the proposed technique’s supremacy from the greenness standpoint.

The greenness profiles generated by the GAPI metric agreed with those generated by the ES metric, proving the proposed technique’s superior greenness to the published techniques.

## Statistical analysis

The findings generated by the proposed potentiometric technique were weighed statistically against those generated by the published technique^15^ for PCPS assay in its crude form. As well, the F-test values (for precision) and t-test ones (for accuracy) were estimated without overriding the referential thresholds (Table S7). Concerning the statistical analysis compiled in Table S7, there aren’t perceptible divergences in functioning between the proposed technique (represented by the proposed three sensors) and the published one, implying the appositeness of the proposed potentiometric technique for regular PCPS assay in QC units.

## Comparison with published approaches

Two different electrochemical approaches^20,21^ for quantifying PCPS in its tablets were identified through a survey of existing research. As detailed in Table S8, our approach’s performance was evaluated against these existing approaches^20,21^. Our approach offers the novel capability of quantifying PCPS in milk and urine samples at very low concentrations, compared to^20,21^. It also demonstrates excellent linearities over wide ranges, as evidenced by high correlation coefficients, compared to^20,21^. Furthermore, our approach utilizes a portable, inexpensive device with simple software, unlike this approach^21^.

## Conclusion

The current study introduces three PVC-based sensors modulated by CDs for rapid in-situ estimation of PCPS in its crude form, commercial product, dissolution media, human urine, and formula milk samples. According to trials, the PCPS-α-CD-PVC sensor produced the optimal response (Nernstian slope) with a wide linear range, quick response time, and long lifespan. The experimental work was backed up by applying molecular docking. The docking study represented by docking scores was consistent with the practical findings represented by slope values, offering a creative strategy to cut costs and time in experimentation. The virtues of high sample throughput with minimal sample pre-treatment, quick analysis, high selectivity, and low detection thresholds make the proposed technique (represented by the proposed three sensors) apt for routine PCPS assessment in QC centers and bioavailability hubs at the lowest expense feasible. Concerning greenness, the proposed technique outperformed the reported technique in this regard.

## Electronic supplementary material

Below is the link to the electronic supplementary material.

Supplementary Material 1

## Data Availability

All data generated or analysed during this study are included in this published article [and its supplementary information files].
