# Supplementary material for: Application of in-silico docking for green electrochemical quantification of prucalopride succinate in pharmaceutical, urine, and milk matrices
Source: Sci Rep. 2025 Feb 18;15:5895. doi: 10.1038/s41598-024-85001-y (PMC11836413; doi:10.1038/s41598-024-85001-y)
Supplement: Supplementary file 1 — Supplementary Material 1 [file 41598_2024_85001_MOESM1_ESM.docx]

**Table S1: Sources and PDB codes of cyclodextrins used in the docking study.**

| **Ionophore** | **PDB code** | **Source** |
| --- | --- | --- |
| α-CD | 3L2M | Pig pancreatic alpha-amylase |
| β-CD | 3CK8 | SusD sugar-binding protein |
| γ-CD | 5MKA | E-coli maltodextrin binding protein |

**Table S2: General features of the various PVC membrane components.**

| **PVC (mg)** | **PCPS-ST ion-pair (mg)** | **Plasticizer (mg)** | **Modifier (mg)** | **Range (M)** | **Slope**^*^ | **LOD (M)** | **Correlation coefficient (r)** |
| --- | --- | --- | --- | --- | --- | --- | --- |
| 190 mg | 8 mg | BA (295 mg) | - | 1×10^-4^ – 1×10^-2^ | 46.42 | 6.60×10^-5^ M | 0.9946 |
| 190 mg | 8 mg | TP (295 mg) | - | 1×10^-5^ – 1×10^-2^ | 49.91 | 6.30×10^-6^ M | 0.9939 |
| 190 mg | 8 mg | DP (295 mg) | - | 1×10^-5^ – 1×10^-2^ | 52.94 | 6.10×10^-6^ M | 0.9989 |
| 190 mg | 7 mg | DP (295 mg) | - | 1×10^-5^ – 1×10^-2^ | 52.80 | 6.80×10^-6^ M | 0.9949 |
| 190 mg | 9 mg | DP (295 mg) | - | 1×10^-5^ – 1×10^-2^ | 52.90 | 6.40×10^-6^ M | 0.9968 |
| **190 mg** | **8 mg** | **DP (295 mg)** | **α-CD (10 mg)** | **1×10^-6^ – 1×10^-2^** | **56.87** | **7.50×10^-7^ M** | **0.9999** |
| 190 mg | 8 mg | DP (295 mg) | β-CD (10 mg) | 1×10^-5^ – 1×10^-2^ | 54.89 | 7.10×10^-6^ M | 0.9998 |
| 190 mg | 8 mg | DP (295 mg) | γ-CD (10 mg) | 1×10^-5^ – 1×10^-2^ | 53.78 | 7.20×10^-6^ M | 0.9997 |

^*^ Mean of three determinations.

**Table S3: Accuracy and precision data obtained for the determination of PCPS by the suggested three sensors.**

| **Sensors**  **Parameters** | **PCPS-α-CD-PVC** | **PCPS-β-CD-PVC** | **PCPS-γ-CD-PVC** |
| --- | --- | --- | --- |
| Accuracy |  | | |
| Mean ± SD | 99.83 ± 1.21 | 99.10 ± 0.96 | 98.64 ± 1.33 |
| RSD% | 1.21 | 0.97 | 1.35 |
| Er%^a^ | -0.17 | -0.90 | -1.36 |
| Intra-day precision^b^ |  | | |
| Mean ± SD | 100.63 ± 0.87 | 99.25 ± 1.42 | 98.97 ± 0.78 |
| RSD% | 0.86 | 1.43 | 0.79 |
| Er%^a^ | 0.63 | -0.75 | -1.03 |
| Inter-day precision^c^ |  | | |
| Mean ± SD | 101.11 ± 1.08 | 100.39 ± 1.26 | 99.05 ± 1.07 |
| RSD% | 1.07 | 1.25 | 1.08 |
| Er%^a^ | 1.11 | 0.39 | -0.95 |

^a^ Mean of three determinations.

^b^ The repeatability; relative standard deviation of (5×10^-5^, 5×10^-4^, 5×10^-3^ M) of PCPS in triplicate within the day using the suggested three sensors.

^c^ The intermediate precision; relative standard deviation of (5×10^-5^, 5×10^-4^, 5×10^-3^ M) of PCPS in triplicate on three consecutive days using the suggested three sensors.

**Table S4: Selectivity coefficients of some interferents using the suggested three sensors.**

| **Sensors**  **Interferent*** | **PCPS-α-CD-PVC** | **PCPS-β-CD-PVC** | **PCPS-γ-CD-PVC** |
| --- | --- | --- | --- |
| Magnesium stearate | 4.25×10^-3^ | 7.26×10^-4^ | 4.63×10^-3^ |
| BaCl_2_ | 7.27×10^-4^ | 6.86×10^-3^ | 4.97×10^-3^ |
| NiCl_2_.6H_2_O | 3.63×10^-3^ | 6.23×10^-3^ | 6.98×10^-4^ |
| KCl | 7.36×10^-4^ | 6.05×10^-3^ | 6.39×10^-3^ |
| NH_4_Cl | 8.24×10^-3^ | 7.18×10^-4^ | 4.35×10^-3^ |
| NaCl | 5.12×10^-3^ | 6.64×10^-3^ | 6.62×10^-4^ |
| Sucrose | 3.08×10^-3^ | 5.67×10^-4^ | 5.70×10^-4^ |
| Glycine | 5.16×10^-4^ | 8.21×10^-4^ | 2.98×10^-3^ |
| Urea | 5.66×10^-3^ | 6.33×10^-3^ | 4.58×10^-3^ |
| Lactose | 3.84×10^-3^ | 3.81×10^-4^ | 7.14×10^-4^ |
| Glucose | 3.39×10^-3^ | 7.25×10^-4^ | 8.40×10^-4^ |
| Starch | 4.53×10^-3^ | 4.51×10^-4^ | 2.31×10^-4^ |
| Microcrystalline cellulose | 2.26×10^-3^ | 4.33×10^-3^ | 3.87×10^-4^ |

* Interferent concentration (1×10^-3^ M).

**Table S5: Robustness study of the suggested three sensors using pure PCPS (5×10^-4^ M).**

| **Variation** | | **PCPS-α-CD-PVC** | **PCPS-β-CD-PVC** | **PCPS-γ-CD-PVC** |
| --- | --- | --- | --- | --- |
|  |  | Recovery%^b^ ± SD | | |
| No variation  (optimum condition)^a^ | | 100.23 ± 0.69 | 99.91 ± 1.32 | 98.79 ± 1.02 |
| Buffer pH | 5.80 | 99.67 ± 1.64 | 100.23 ± 0.87 | 99.77 ± 0.67 |
|  | 6.20 | 101.68 ± 1.28 | 101.02 ± 0.72 | 100.60 ± 1.12 |
| Soaking time | 23 h | 99.23 ± 0.98 | 99.07 ± 1.35 | 99.80 ± 1.61 |
|  | 25 h | 100.68 ± 0.91 | 98.92 ± 1.43 | 98.30 ± 1.32 |

^a^ Optimum condition: buffer pH (pH=6) and soaking time (24 h).

**^b^** Mean of three determinations.

**Table S6: Results of eco-scale analysis for the determination of PCPS employing the suggested potentiometric approach and the reported [15,20,21] approaches.**

| **Approaches**  **Parameters** | **Suggested approach** | **Reported LC [15] approach** | **Reported [20] approach** | **Reported [21] approach** |
| --- | --- | --- | --- | --- |
| **Reagents** |  | | | |
| Methanol | – | 12 | – | – |
| Acetonitrile | – | 8 | – | – |
| Ammonium formate buffer | – | 0 | – | – |
| Polyvinyl chloride | 0 | – | – | – |
| Sodium tetraphenylborate | 0 | – | – | – |
| Tetrahydrofuran | 6 | – | – | – |
| Cyclodextrins | 0 | – | – | – |
| Dioctyl phthalate | 0 | – | – | – |
| Graphite powder | – | – | 1 | 1 |
| Zeolite | – | – | 1 | – |
| Phosphotungstic acid | – | – | 2 | – |
| Dibutyl phthalate | – | – | 0 | – |
| Paraffin oil | – | – | – | 2 |
| ZrO_2_ nanoparticles | – | – | – | 3 |
| Ethyl ether | – | – | – | 2 |
| Phosphate buffer | 0 | – | – | 0 |
| Britton-Robinson buffer | 0 | – | – | – |
| **Instruments** |  | | | |
| Energy | 0  [≤ 0.1 kWh/sample] | 1  [> 0.1 kWh/sample] | 0  [≤ 0.1 kWh/sample] | 0  [≤ 0.1 kWh/sample] |
| Occupational hazard | 0 | 3 | 0 | 0 |
| Waste | 0 | 5 | 3 | 3 |
| Total penalty points | Σ 6 | Σ 29 | 7 | 11 |
| Analytical eco-scale total score^a,b^ | 94 | 71 | 93 | 89 |
|  | Excellent green analysis | Acceptable green analysis | Excellent green analysis | Excellent green analysis |

^a^ Analytical eco-scale total score = 100- total penalty points.

^b^ If the score is > 75, it signifies excellent green analysis.

If the score is > 50, it signifies acceptable green analysis.

If the score is < 50, it signifies inadequate green analysis.

**Table S7: Statistical analysis of the suggested potentiometric approach and the reported LC approach [15] for the determination of PCPS in its crude form.**

| **Parameters** | **Suggested sensors** | | | **Reported LC approach** |
| --- | --- | --- | --- | --- |
|  | **PCPS-α-CD-PVC** | **PCPS-β-CD-PVC** | **PCPS-γ-CD-PVC** |  |
| **N** | 5 | 5 | 5 | 5 |
| **Mean** | 100.25 | 99.34 | 98.98 | 100.32 |
| **SD** | 1.13 | 0.98 | 1.10 | 1.20 |
| **Variance** | 1.28 | 0.96 | 1.21 | 1.44 |
| **Student’s *t*-test**^a^  **(2.31)** | 0.09 | 1.41 | 1.84 | **——** |
| ***F*-value**^a^  **(6.39)** | 1.13 | 1.50 | 1.19 | **——** |

^a^ The values in parenthesis are tabulated values of “*t* ” and “*F* ” at (*P* = 0.05).

**Table S8: Comparing our suggested potentiometric approach with other published electrochemical approaches.**

| **Approach** | **Linearity range (M)** | **Correlation coefficient (r)** | **Applications** | **References** |
| --- | --- | --- | --- | --- |
| Potentiometry | 2×10^-6^ – 1×10^-2^ | 0.9988 | Tablets | **[20]** |
| Voltammetry | 4.03×10^-7^ – 2.20×10^-6^ | 0.9992 | Tablets | **[21]** |
| Suggested  potentiometry | 1×10^-6^ – 1×10^-2^ | 0.9999 | Tablets | **——** |
|  | 1×10^-6^ – 1×10^-4^ | 0.9996 | Urine | **——** |
|  | 1×10^-6^ – 1×10^-4^ | 0.9997 | Milk | **——** |


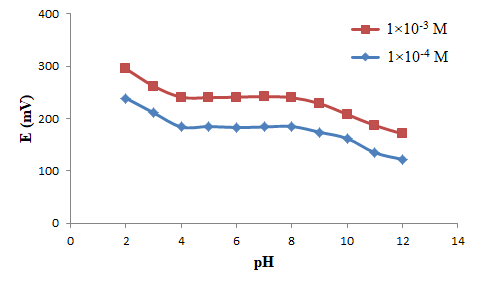

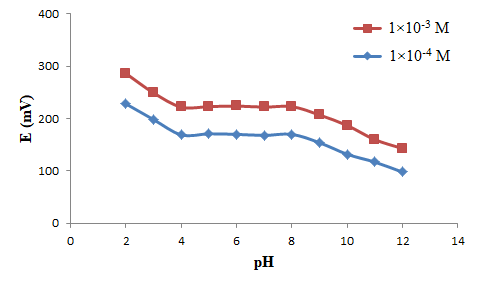


**(a) (b)**

**
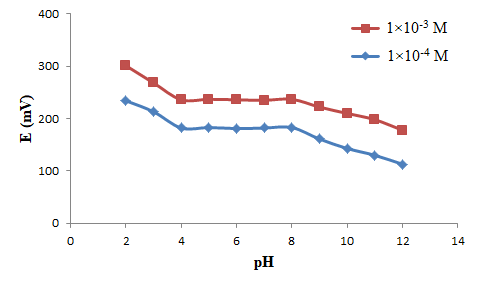
**

**(c)**

**Figure S1:** The influence of pH on the performance of **(a)** PCPS-α-CD-PVC sensor, **(b)** PCPS-β-CD-PVC sensor, and **(c)** PCPS-γ-CD-PVC sensor using PCPS solutions (1×10^-3^ and 1×10^-4^ M).


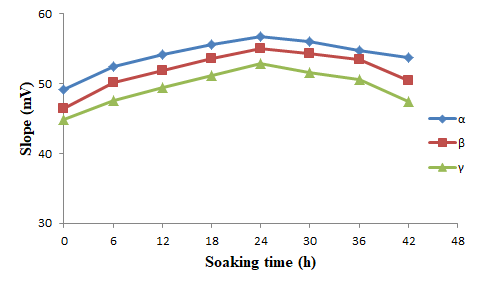


**Figure S2:** Soaking time impact on the behavior of the suggested three sensors.


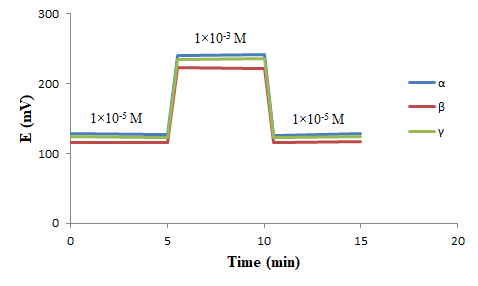


**Figure S3:** The reversibility of the suggested three sensors after exposure to different concentrations of PCPS.


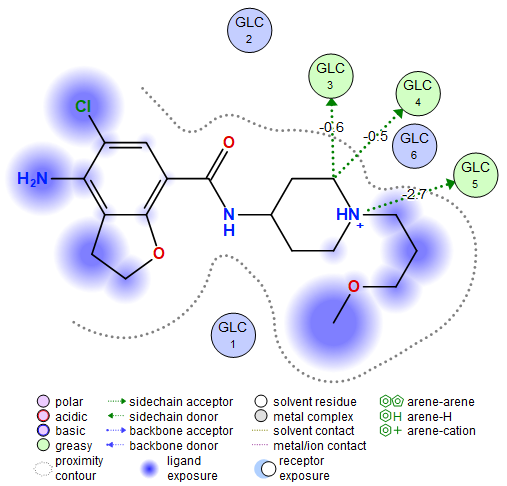

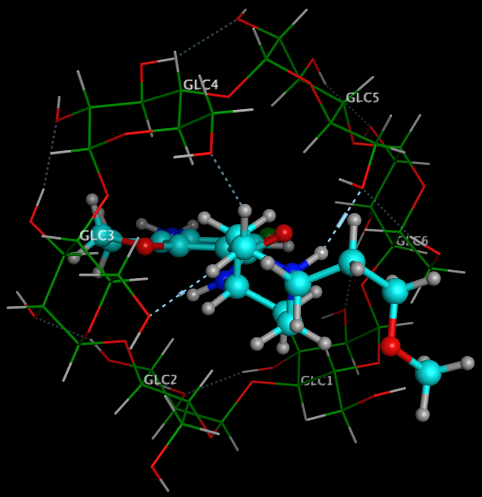


**2D**

**3D**

**(a)**


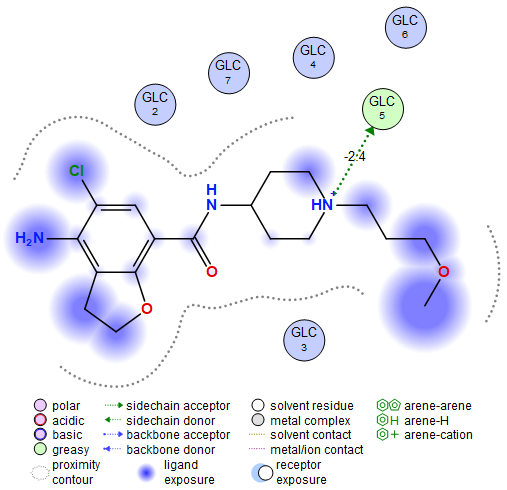

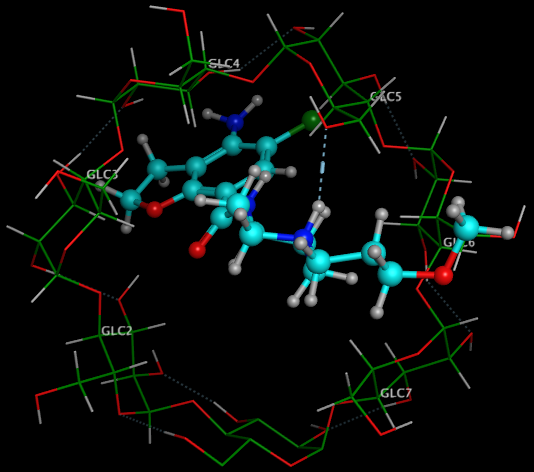


**3D**

**2D**

**(b)**


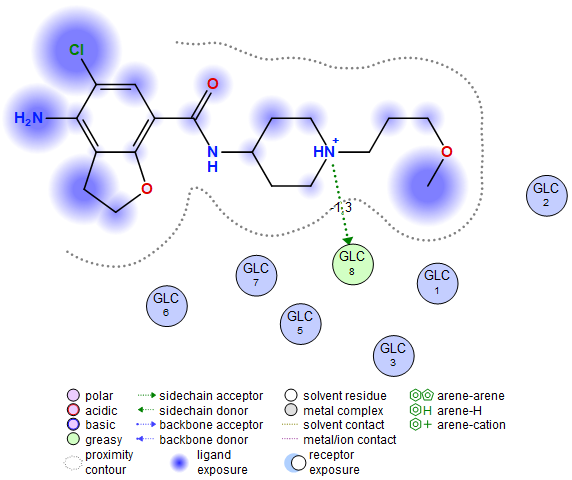

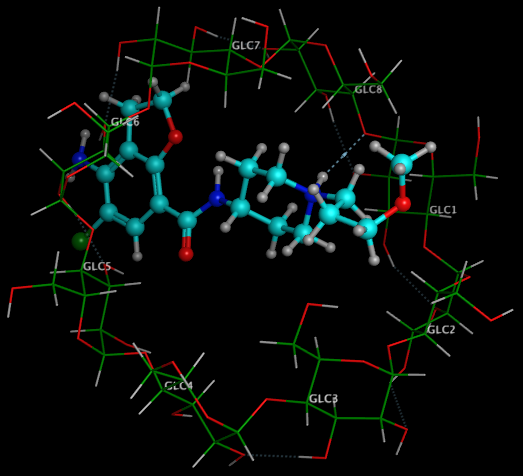


**3D**

**2D**

**(c)**

**Figure S4:** Molecular interactions between PCPS and **(a)** α-CD, **(b)** β-CD, and **(c)** γ-CD.

**
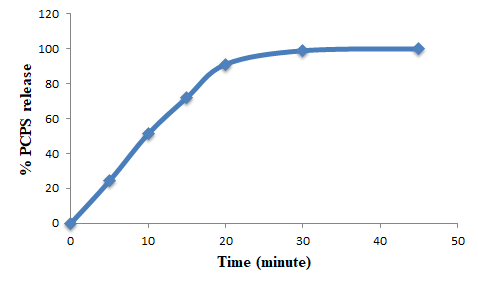
**

**Figure S5:** In-vitro dissolution profile of Prucasoft^®^ tablets using the suggested sensor (PCPS-α-CD-PVC).
